# The Generation of the Oxidant Agent of a Mononuclear Nonheme Fe(II) Biomimetic Complex by Oxidative Decarboxylation. A DFT Investigation

**DOI:** 10.3390/molecules25020328

**Published:** 2020-01-14

**Authors:** Angela Parise, Maria Costanza Muraca, Nino Russo, Marirosa Toscano, Tiziana Marino

**Affiliations:** Dipartimento di Chimica e Tecnologie Chimiche, Università della Calabria, Cubo 14C, Via P. Bucci, 87036 Arcavacata di Rende, CS, Italy; angela.parise@unical.it (A.P.); muraca.mc@gmail.com (M.C.M.); marirosa.toscano@unical.it (M.T.)

**Keywords:** nonheme biomimetic, potential energy surface, density functional theory, oxidative decarboxylation

## Abstract

The oxidative decarboxylation of the iron(II) α-hydroxy acid (mandelic acid) complex model, biomimetic of Rieske dioxygenase, has been investigated at the density functional level. The explored mechanism sheds light on the role of the α-hydroxyl group on the dioxygen activation. The potential energy surfaces have been explored in different electronic spin states. The rate-determining step of the process is the proton transfer. The oxidative decarboxylation preferentially takes place on the quintet state.

## 1. Introduction

In recent years, the employment of biomimetic catalysts considerably increased not only for their related high selectivity, but because they are allowed to operate in very mild conditions and they adapt to different types of substrates [1,2].

Encouraged by the new reactions catalyzed by Rieske dioxygenases [1,3,4], sustainable mononuclear nonheme iron complexes have been proposed for bioinspired oxidation catalysis. Dioxygenases are involved in the degradation of a wide range of natural and synthetic compounds by incorporating both atoms of O_2_ into substrates.

**Scheme 1:**
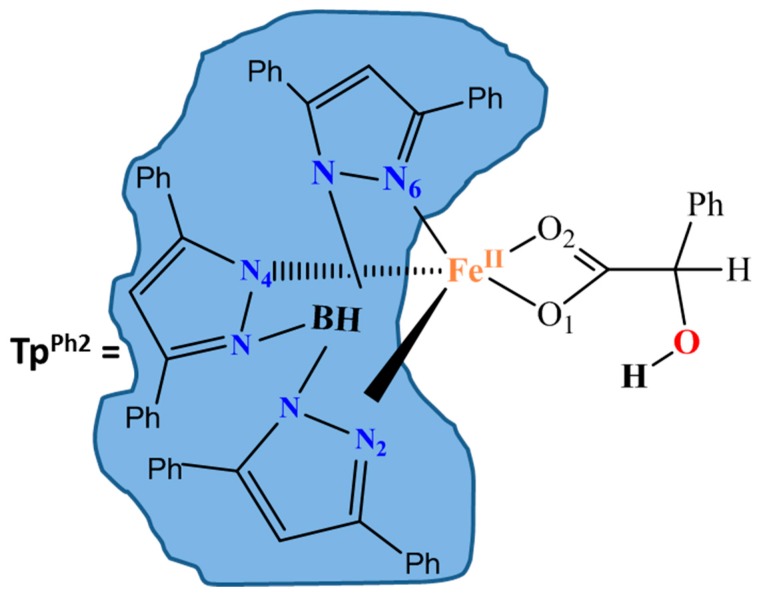
Structure of hydrotris(3,5-diphenylpyrazole-1-yl)borate FeII (mandelate).

Bioinspired catalysis using peroxides or peracids for the oxidation of hydrocarbon substrates is more common and better characterized, than that based on dioxygen in catalytic oxidations, which has not been fully explored yet and the relative reaction mechanism is often unknown. This last reaction path requires both electron and proton sources [5,6,7,8,9,10,11,12,13] and cosubstrate-assisted O_2_ molecular activation by transition metal containing complexes can be an alternative pathway [14,15,16,17,18,19] resorting to the sacrificial reductants represented a good solution [12,20]. Paine’s and other groups have investigated a family of iron complexes supported by the monoanionic facial trinitrogen donor ligand, hydrotris(3,5-diphenylpyrazole-1-yl)borate (Tp^Ph2^ ligand) that activates O_2_ by 2 electrons reduction in the oxidative decarboxylation of the α-hydroxy acids as sacrificial reductants [3,21,22,23]. These studies provide evidence that the the hydroxyl group in the dioxygen activation is important. The iron complexes mentioned above reacting with O_2_ are able to carry out the stoichiometric cis-dihydroxylation of olefins producing the diol product [3], as in that with H_2_O_2_ [4,24,25]. The mononuclear non-heme iron(II) complex shown in Scheme 1, the object of the present investigation, represents the first biomimetic complex reproducing the dioxygenase activity of α-ketoglutarate-dependent enzymes. 

Since, as occurred in similar systems [26] no intermediate was detected in the course of the reaction, the steps up to the oxidative decarboxylation of the keto acid play a key role in the control of the rate of the reaction.

To provide deeper insights on the reaction catalyzed by the biomimetic complex of Scheme 1, we have undertaken a theoretical investigation taking into account the spin state of the analyzed species and building up the potential energy surfaces for the reaction path leading to the formation of an iron−oxygen oxidant upon oxidative decarboxylation of α-hydroxy acid.

## 2. Results and Discussion

As a first step of work, we have optimized the naked biomimetic iron(II) α‑hydroxy acid complex (represented by model **a** in Figure S1 of the Supplementary Information) in septet, quintet, triplet and singlet spin states.

The coordination bond distances, which we have obtained together with X-ray counterpart [3] and relative electronic energies are collected in Table 1. Results show that the complex assumes the most stable configuration in the high-spin quintet state in well agreement with the available data for similar systems [3,27,28]. The singlet, triplet and septet spin states lie at 17.2, 13.1, and 65.1 kcal/mol above ground one, respectively.

Comparison with the crystallographic structure (Table 1) shows that the geometrical parameters in the quintet state are reproduced fairly faithfully. Furthermore, the calculated Fe-N bond distances well match with other high-spin (Tp^Ph2^) Fe^II^ complexes [23,27].

**Figure 1:**
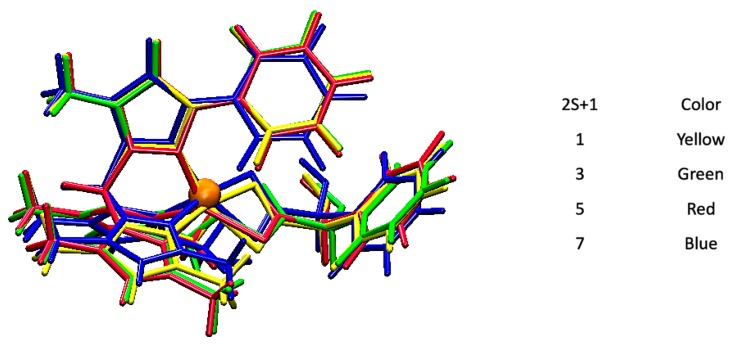
Superposition of the M06L optimized structures of model **a** at the different spin states considered.

The maximum deviation results to be less than 0.06 Å. From the superposition of the optimized structures at the considered spin states, it emerges that the mandelate ring shows major deviations whereas the coordination around the iron does not suffer drastic changes (Figure 1). This suggests that in the quintet, the carboxylic group of the mandelic acid acts as monodentate ligand.

**Figure 2:**
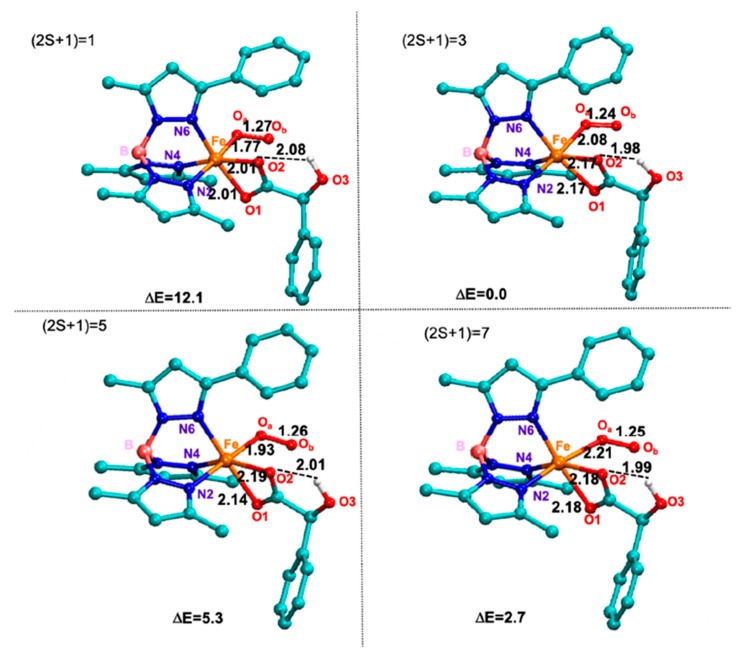
M06L optimized structures and main geometrical parameters of **a**-O_2_ adducts. Selected bonds are in Å. Relative electronic energies are in kcal/mol.

**Figure 3:**
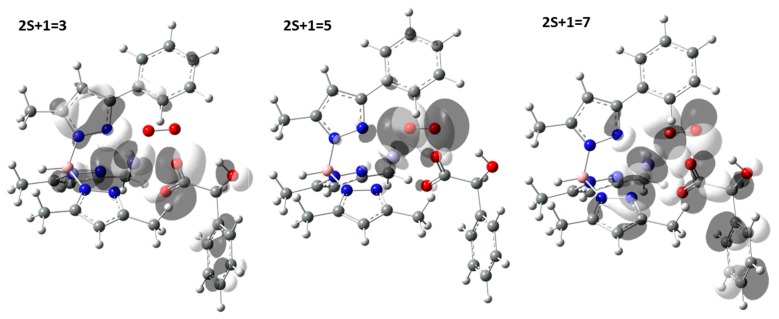
Highest occupied molecular orbital (HOMO) composition of **a**-O_2_ adduct, in the triplet, quintet and septet states.

For the O_2_ addition (**a**-O_2_ complex), two different binding modes, namely end-on (eo) and side-on (so), have been taken into account. From computations, the eo binding mode appears to be the preferred one in all the considered spin states. Looking at the Figure 2 collecting the optimized structures of the eo obtained adducts, the triplet **a**-O_2_ complex represents the lowest energy configuration followed by the septet, quintet and singlet. A support to this finding arises from the analysis of the triplet spin state HOMO frontier orbital (see Figure 3) that mainly involves the metal center and its surrounding with the mandelate ligand less interested in the occurring electron redistribution.

As concerns the geometrical structure, the entry of dioxygen induces a reorganization around the metal center that now appears to be hexacoordinated (octahedral geometry). The O_2_ occupies the apical position, while the carboxylate moiety (lying on the equatorial plane) acts as bidentate ligand with both the Fe-O bond lengths of 2.17 Å. These values well compare with those present in dioxygenase enzymes [20,26].

The Fe-O_a_ bond assumes the value of 2.08, 1.93, and 2.21 Å in the triplet, quintet and septet electronic states, respectively. The O-O bond distance in the ground state (1.24 Å) reveals a significant deviation from the corresponding value in the enzymatic environment (1.31 Å). This behavior can be ascribable to the weaker electro-donating character of Tp^Ph2^ ligand towards the iron(III), present in the **a**-O_2_ complex, relative to the usual biological ligands in the enzymes [26].

**Scheme 2:**
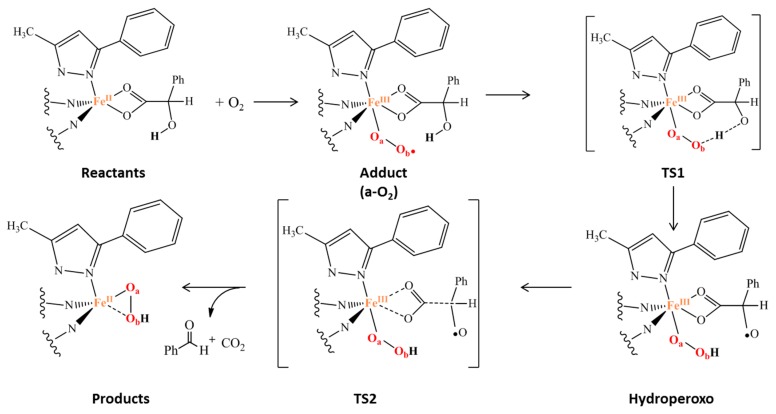
Proposed mechanism for the oxidative decarboxylation promoted by the non-heme biomimetic complex model (**a**).

The considered reaction mechanism (Scheme 2) arises from experimental indications. In fact, biomimetic and enzymatic studies suggested that the iron(III) superoxide species, obtained upon addition of O_2_ at the iron(II) center, has the ability to abstract hydrogen atom from the O-H group of α-hydroxy acid.

**Figure 4:**
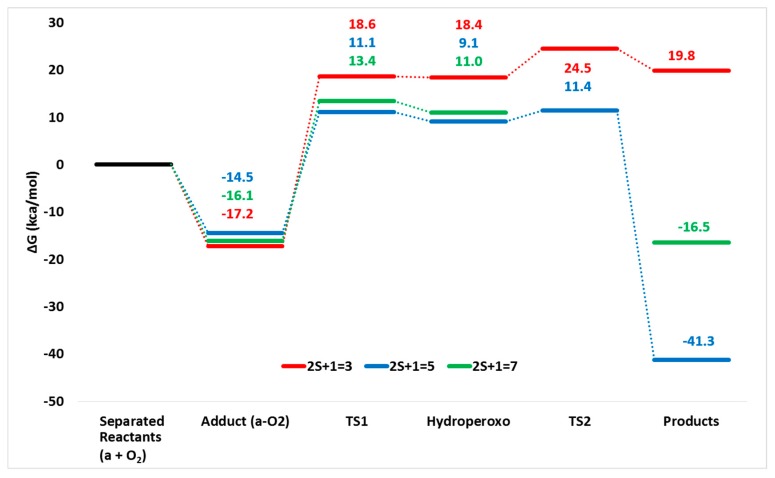
M06L potential energy surfaces (PESs) for oxidative decarboxylation operated by non-heme biomimetic model (**a**) at different spin states (triplet, quintet and septet).

Since the singlet electronic spin state of iron-dioxygen complexes lies at very high energy, only the septet, quintet and triplet potential energy surfaces (PESs) have been considered (see Figure 4).

As far as the PES for the septet is concerned, no transition state connecting the hydroperoxide with the final oxidant agent (TS2 of Scheme 2) has been intercepted along the surface. A similar finding has been previously obtained for two enzymatic systems [26,29]. Instead, on the quintet and triplet PESs, both TS1 and TS2 have been characterized confirming that the oxidative decarboxylation is a multistep process.

The addition of O_2_ molecule starting to the complex (**a**) leads to the formation of adducts that in all the three considered spin states lie below the reactants’ asymptote suggesting as their formation is an exergonic process (see Figure 4).

**Figure 5:**
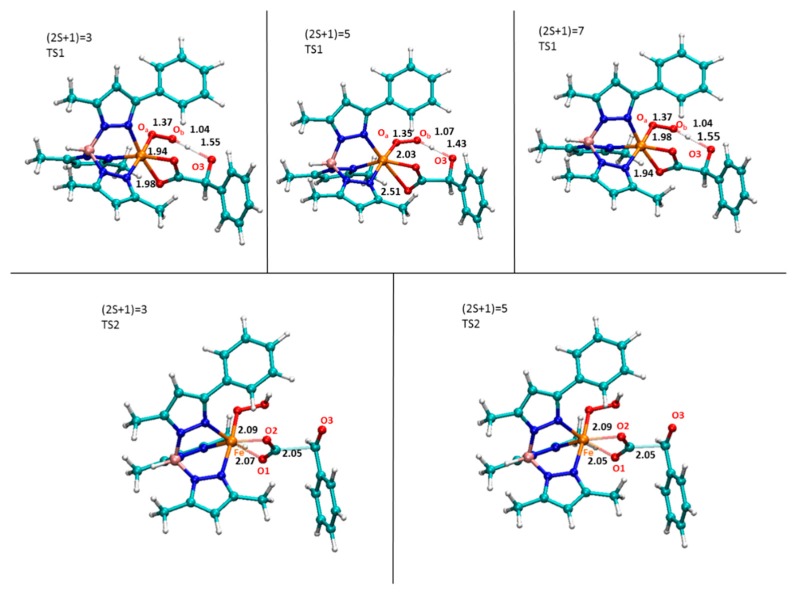
M06L optimized structures of TS1 and TS2 intercepted along the three explored PESs. Selected bonds are in Å.

On the three computed potential energy surfaces, the first transition state (TS1) is located at 18.6 (triplet), 11.1 (quintet) and 13.4 (septet) kcal/mol and their corresponding optimized structures are shown in Figure 5.

For the TS1 in the triplet state, the barrier is 7.5 kcal/mol higher than that of quintet and a spin crossing from these two PESs occurs. This is due to the fact that the resulting iron(III) hydroperoxo species prefers to be a quintet (9.1 kcal/mol), while the corresponding in the triplet state is 9.3 higher in energy. The TS1 describes the proton transfer from the α-OH group of the mandelate to the O_b_ atom of the molecular oxygen, as confirmed by the imaginary frequency (638.1*i* cm^−1^). This result enhances the role played by the α-hydroxy acid ligand in this process as proposed by Paine et al. [3].

**Figure 6:**
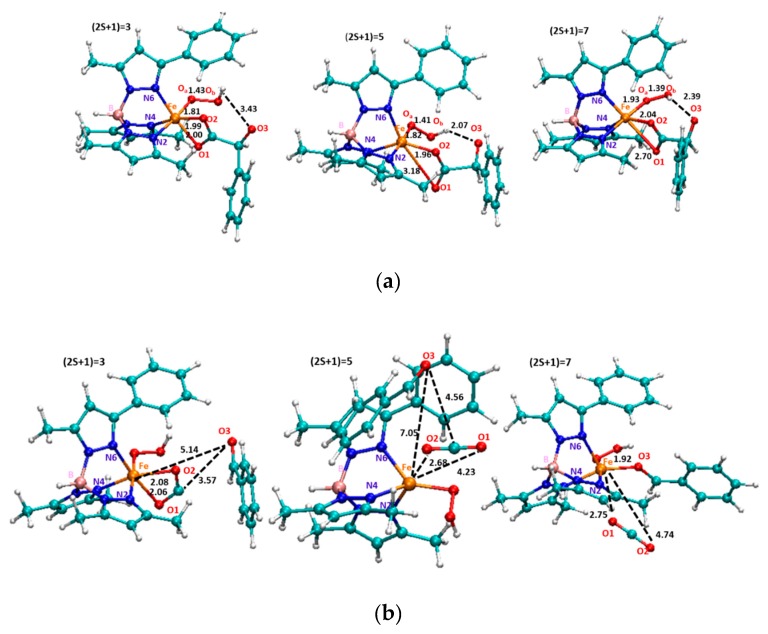
M06L optimized structures and main geometrical parameters of hyproperoxo (**a**) and prod species (**b**) in all the three electronic states considered. Selected bonds are in Å.

The next formed species, containing the Fe(III)-OOH moiety, is found at 9.1 kcal/mol and 11.0 kcal/mol in the quintet and septet state, respectively. In contrast, the iron(III) hydroperoxo in the triplet state lies at higher energy. This finding reflects the relative geometrical differences (see Figure 6a) In fact, in the quintet state, the carboxylate moiety is monocoordinated to iron (O_1_-Fe: 1.96 Å, O_2_-Fe: 3.18 Å) and the C-C bond results to be more elongated (1.71 Å) with respect to the previous steps. The benzaldehyde is prone to be released but it still retained by an H bond between its carbonyl (2.07 Å) and the OOH moiety. From the iron(III) hydroperoxo, the reaction proceeds with the definitive C-C bond breaking (2.05 Å) via TS2 that is characterized by an imaginary frequency related to the C-C stretching mode (211.8*i* cm^−1^) as shown in Figure 4. In addition, this bond’s elongation generates the loss of the H bond since the OH-O=C is now 3.35 Å.

The product in its quintet spin state, at 41.3 kcal/mol below the separated reactants, evidences as the decarboxylation is a highly exergonic process. In the product, the CO_2_ is already formed (O-C-O angle is 180°) and it lies at 2.67 Å from the metallic center engaged in a sandwich-like topology between two phenyl rings (ligand and benzaldehyde) (see Figure 6b). Some changes occur also in the metallic core due to the elongation of O_a_-O_b_ bond (1.48 Å) and to the Fe-O_a_ distance of 1.91 Å. In this way, the iron(II) hydroperoxo oxidant agent is formed and ready to act as nucleophilic agent in the next oxidation reactions that are not object of the present study.

Paine et al. underline as “this iron(II) hydroperoxo species may also undergo O-O bond heterolysis to form an iron(IV)-oxo-hydroxo intermediate and both oxidant species can carry out the oxidation on different kind of substrates” [3]. To give a contribution to this aspect, geometry optimizations of the two forms have been performed and the resulting optimized geometries (shown in Figure S2) indicate that the quintet spin state proves to be the most stable one.

## 3. Conclusions

On the basis of our density functional theory investigation on the oxidative decarboxylation promoted by a biomimetic nonheme complex, the following conclusions can be drawn:

1. The favored reaction path occurs in the quintet spin state as in the corresponding Rieske oxygenase enzymes;
2. A spin cross takes place between the triplet and quintet electronic spin states after the adduct formation;
3. The α-hydroxy group plays an important role in the oxidative decarboxylation;
4. The rate determining step is the proton transfer;
5. The overall reaction results to be more exergonic in the quintet PES.

## 4. Computational Details

All density functional theory (DFT) based calculations have been performed by using Gaussian09 code [30]. Following previous suggestions available in literature about the reliable results of the M06L [31,32] exchange-correlation functional in describing electronic structure and properties of metal containing systems also in various spin states [33,34,35,36], this functional has been adopted for the geometry optimizations.

The crystal structure (X ray) of the biomimetic catalyst [3] has been adopted as starting structure for our computations. In order to reduce the computational time, five phenyl rings of the Tp^Ph2^ ligand have been replaced by methyl groups (see **a** of Figure S1). The phenyl group of the Tp^Ph2^ ligand closely oriented to the iron center has been retained to better evaluate its role on the activated dioxygen species during the catalytic process.

Geometry optimizations and vibrational frequencies have been performed employing differentiated basis set. In particular, for O, N and H atoms in the active center the 6-31+G(d,p) set has been used while for the remaining ones the 6-31G(d,p) one has been chosen. The LANL2DZ effective core potential and the related basis set has been employed for iron.

In order to improve the energetic values, single point calculations with the larger basis set 6-311+G(d,p) for all atoms except for metal ion, has been performed on all the previously optimized structures. The solvent corrections were evaluated using the polarizable continuum model [37,38] as implemented in Gaussian09 taking into account the dielectric constant (ε = 24.93) that better reproduces the polarity of the solvents mixture used in the experimental work [3].

Owing to the unrestricted formalism of all calculations, the spin contamination and its effect on the calculated energetics was monitored as done for similar systems [35,39] and the <S2> values are collected in Supplementary Information section (Table S1).

## Figures and Tables

**Table 1:** Main geometrical parameters for naked complex **a** at all the spin states. Relative energies provided at M06L level of theory are kcal/mol and compared with the experimental X-ray counterpart [3]. Distances are in Å. See Scheme 1 for labels.

Bond	2S + 1				X-Ray
	1	3	5	7	
Fe-O1	2.06 Å	2.04 Å	2.01 Å	2.06 Å	2.01 Å
Fe-O2	2.08 Å	2.03 Å	2.19 Å	2.29 Å	2.17 Å
Fe-N2	1.96 Å	2.18 Å	2.10 Å	2.09 Å	2.17 Å
Fe-N4	1.96 Å	1.95 Å	2.16 Å	2.05 Å	2.10 Å
Fe-N6	1.92 Å	2.00 Å	2.09 Å	1.97 Å	2.11 Å
DE	17.2	13.1	0.0	65.1	-

## Supplementary Materials

Supplementary materials can be found online.

## Abbreviations

Tp^Ph2^	hydrotris (3,5-diphenylpyrazole-1-yl) borate
DFT	Density Functional Theory
PES	Potential Energy Surface
PCM	Polarizable Continuum Model
HOMO	High Occupied Molecular Orbital
