# Supplementary material for: The Generation of the Oxidant Agent of a Mononuclear Nonheme Fe(II) Biomimetic Complex by Oxidative Decarboxylation. A DFT Investigation
Source: Molecules. 2020 Jan 14;25(2):328. doi: 10.3390/molecules25020328 (PMC7024176; doi:10.3390/molecules25020328)
Supplement: Supplementary file 1 [file molecules-25-00328-s001.pdf]

Supplementary Information

The generation of the oxidant agent of a mononuclear nonheme Fe(II) biomimetic complex by oxidative decarboxylation. A DFT investigation

Angela Parise, Maria Costanza Muraca, Nino Russo, Marirosa Toscano and Tiziana Marino,

Dipartimento di Chimica e Tecnologie Chimiche, Università della Calabria, Cubo 14C, Via P. Bucci, 87036 Arcavacata di Rende, CS, Italy;

Table S1. Calculated spin contamination for all investigated complexes under all three spin states co spin states examined.

	**3**	**5**	**7**
**a**	2.0442	6.0127	12.0233
adduct	3.5769	6.6643	12.0201
TS1	2.0210	6.6945	12.0188
hydroperoxo	2.0294	6.1325	12.0182
TS2	2.0361	6.1380	-
Prod	2.0334	6.0156	12.0297

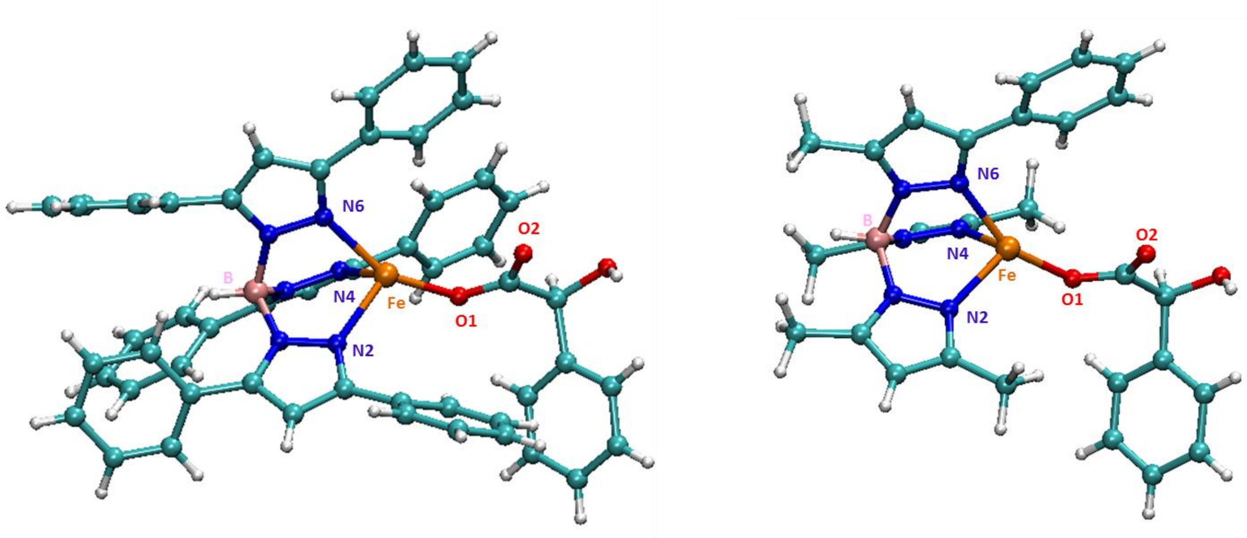

X-Ray

Model
(a)

Figure S1 Crystal structure of hydrotris(3,5-diphenylpyrazole-1-yl)borate) Fe(II)-mandelate and its computational model (**a**) used in this study.

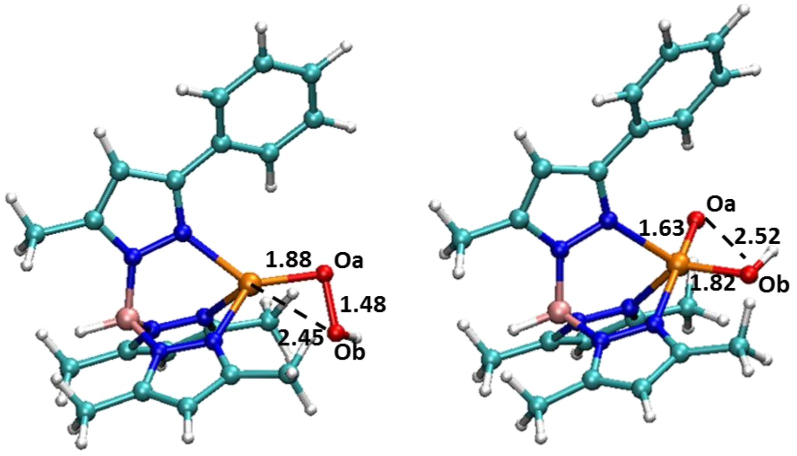

(I)
Iron (II) –hydroperoxo species
36.1 kcal/mol

(II)
iron(IV)–oxo–hydroxo species
0.0 kcal/mol

Figure S2 The two possible forms of the product of the oxidative decarboxylation process in quintet species.
